# Association between Creatinine Trajectories and All-Cause Mortality in Elderly Patients after Cardiac Surgery: A Retrospective Cohort Study Based on MIMIC-IV

**DOI:** 10.5761/atcs.oa.26-00016

**Published:** 2026-04-03

**Authors:** Yujie Fan, Zefeng Yang, Jiayao Wei, Qiang Li

**Affiliations:** Department of Cardiothoracic Surgery, The Second Hospital of Shanxi Medical University, Taiyuan, China

**Keywords:** creatinine, mortality, cardiac surgery, trajectory analysis

## Abstract

**Purpose:**

Conventional monitoring of serum creatinine at isolated time points is suboptimal for reflecting disease progression. We therefore evaluated the prognostic value of distinct serum creatinine trajectories for predicting all-cause mortality in this patient population.

**Methods:**

We conducted a retrospective cohort study of elderly patients undergoing cardiac surgery, using data from the Medical Information Mart for Intensive Care database (MIMIC-IV). Kaplan–Meier analysis compared survival outcomes among the identified classes. Multivariable logistic and Cox proportional hazards models determined the independent associations of these trajectories with in-hospital and 1-year all-cause mortality.

**Results:**

A total of 5145 patients undergoing cardiac surgery were included in the analysis. Three distinct creatinine trajectories were identified: low-stable (Class 1), persistent-increase (Class 2), and transient-increase (Class 3). Class 1 had the lowest in-hospital and 1-year mortality rates. Using Class 1 as the reference, Class 2 demonstrated the highest mortality risk, followed by Class 3.

**Conclusion:**

Distinct creatinine trajectory patterns conferred independent prognostic value for postoperative outcomes in elderly patients after cardiac surgery. A persistently high trajectory was independently associated with significantly increased mortality compared to the low-stable trajectory class.

## Introduction

Due to population aging, cardiovascular diseases have become increasingly prevalent, leading to a growing demand for cardiac surgery. Hemodynamic changes during surgery can trigger neurohormonal adaptive responses, which may subsequently result in reduced renal perfusion.^1)^ Patients with better outcomes often exhibit lower creatinine levels,^2)^ potentially attributable to early and aggressive fluid resuscitation that alleviates renal hypoperfusion and preserves glomerular filtration.^3)^ Higher mortality rates following cardiac surgery are associated with more complex or combined procedures and high-risk patient profiles.^4,5)^

A mild elevation in creatinine levels was associated with increased 90-day mortality and prolonged hospitalization.^6)^ Even a slight rise in creatinine was directly linked to adverse outcomes.^7–10)^ Acute kidney injury (AKI) is attributed to multiple factors, such as hypoperfusion, ischemia–reperfusion injury, inflammatory responses, oxidative stress, and exposure to nephrotoxins.^11)^ The incidence and severity of AKI vary significantly across surgical types, with the lowest rate observed in coronary artery bypass grafting (CABG) (19%), and higher rates in valve surgery (27.5%) or aortic procedures (29%).^12,13)^

## Materials and Methods

### Data source

The Medical Information Mart for Intensive Care IV (MIMIC-IV) database (https://mimic-iv.mit.edu/) was used to collect the study data. MIMIC-IV is a large-scale, single-center public database focused on critical care within the US healthcare system. It comprises electronic health record data collected between 2008 and 2019 by Beth Israel Deaconess Medical Center in Boston, Massachusetts. The database contains detailed clinical information for over 300000 hospital admissions and is made available to researchers worldwide after de-identification. This initiative does not affect clinical care, and the patients in the database cannot be identified; therefore, individual patient consent or ethical approval is not required.^14)^ This study adheres to the guidelines of the Strengthening the Reporting of Observational Studies in Epidemiology (STROBE) statement^15)^ and complies with the principles outlined in the Declaration of Helsinki.

### Patients

A total of 8560 elderly patients who underwent cardiac surgery were initially identified from the MIMIC-IV database using International Classification of Diseases (ICD) codes. Patients were excluded if they met any of the following criteria: 1) lack of multiple serum creatinine measurements within the first 4 days post-surgery; 2) missing survival status information; or 3) a postoperative hospital stay of less than 4 days.

### Observational variables

The primary study variable was the creatinine recorded within the first 4 days following surgery. Additional variables included: (1) age, sex, and race; (2) the first laboratory values and vital signs obtained within 24 h after cardiac surgery: International Normalized Ratio (INR), prothrombin time (PT), partial thromboplastin time (PTT), chloride, bicarbonate, blood urea nitrogen (BUN), potassium, white blood cell count (WBC), mean corpuscular hemoglobin concentration (MCHC), red cell distribution width (RDW), red blood cell count (RBC), platelets, mean corpuscular volume (MCV), mean corpuscular hemoglobin (MCH), hemoglobin, hematocrit, lactate, pH, oxygenation index (PaO_2_/FiO_2_ ratio), partial pressure of carbon dioxide (pCO_2_), fibrinogen, thrombin, albumin, calcium, glucose, heart rate, respiratory rate, systolic blood pressure (SBP), diastolic blood pressure (DBP), and oxygen saturation (SpO_2_); (3) preoperative comorbidities, including myocardial infarction, diabetes mellitus, congestive heart failure (CHF), and cerebrovascular disease (CVD); (4) postoperative supportive therapies, such as norepinephrine, dopamine, vasopressin, and continuous renal replacement therapy; (5) extracorporeal circulation auxiliary to open heart surgery (defined as the use of cardiopulmonary bypass during the procedure); (6) outcome variables, including in-hospital, 30-day, 180-day, and 1-year all-cause mortality after admission. Data were extracted from the MIMIC-IV database using Navicat Premium 17.0 and Structured Query Language (SQL).

### Data processing

Outliers in creatinine data were corrected using Winsorization. Variables with a missing rate exceeding 20% for laboratory measures or covariates were excluded from the analysis. For variables with a missing rate below 20%, multiple imputations were applied to handle missing data.

### Statistical analysis

Continuous variables are presented as mean ± standard deviation (SD) or median with interquartile range (IQR), as appropriate. Categorical variables are summarized as numbers and percentages (n, %). Class comparisons were performed using the Wilcoxon rank-sum test for continuous variables and the chi-squared or Fisher’s exact test for categorical variables. The association between creatinine trajectories and survival over time was visualized using Kaplan–Meier curves. Cox proportional hazards regression models were employed to quantify the impact of creatinine trajectories on survival outcomes. Three sequentially adjusted models were constructed: Model 1 included only the creatinine trajectory variable. Model 2 was further adjusted for key demographic characteristics (age, gender, and race). For Model 3, we first performed univariate screening to identify potential predictors of 1-year mortality (significance level set at p <0.05). Following this screening, significant variables—including vital signs (heart rate, respiratory rate, diastolic blood pressure), laboratory parameters (hemoglobin, prothrombin time, partial thromboplastin time, platelet count, white blood cell count, lactate, and partial pressure of carbon dioxide), and comorbidities (congestive heart failure, myocardial infarction, cerebrovascular disease, and diabetes)—were incorporated into the final multivariable model, along with the demographic covariates from Model 2.

To explore the heterogeneity of postoperative creatinine changes among patients, we employed a method known as trajectory analysis. This approach automatically classifies patients with similar patterns of change based on their serial postoperative creatinine measurements. By identifying these distinct trajectory classes, we can more precisely characterize the evolution of postoperative creatinine values and provide richer information for clinical prognostication, rather than relying solely on creatinine values at a single time point. To determine the optimal number of latent classes, we estimated models with 2 to 7 trajectories. Model fit was assessed using the log-likelihood, entropy, Akaike information criteria (AIC) and Bayesian information criteria (BIC), with lower AIC and BIC values indicating a better fit. An entropy value above 0.7 was considered indicative of high classification accuracy. To ensure model stability, we required each class to comprise at least 2% of the sample and the average posterior probability for all classes to be ≥0.7.

## Results

### Trajectory classification based on the LCTM model

**Fig. 1:**
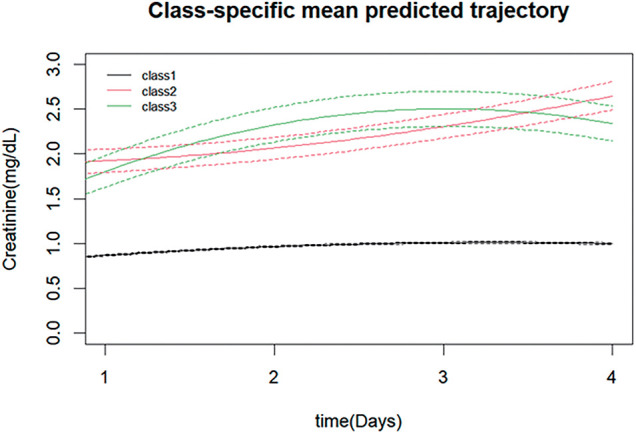
Three distinct creatinine trajectories identified by LCTM over the first 4 postoperative days. Solid lines represent class-specific mean predicted trajectories, with dotted lines indicating the corresponding 95% confidence intervals. Class 1 (“low-stable”): maintained stable creatinine levels around 0.9 mg/dL throughout days 1–4; Class 2 (“persistent-increase”): presented with initial creatinine of ~1.9 mg/dL on day 1 with continued linear increase to ~2.4 mg/dL by day 4; Class 3 (“transient-increase”): began at ~1.7 mg/dL on day 1, peaked around day 2–3 (~2.5 mg/dL), followed by gradual recovery toward baseline by day 4. LCTM: latent class trajectory modeling

The creatinine trajectories identified by Latent Class Trajectory Model (LCTM) are shown in **Fig. 1**. The model stratified the development cohort into 3 distinct classes: Class 1 (n = 4408, 85.68%), Class 2 (n = 472, 9.17%), and Class 3 (n = 265, 5.15%). Class 1 (“low-stable”) maintained a stable mean creatinine level around 0.9 mg/dL; this stable low-level trajectory likely reflects preserved overall physiological function without significant impairment, suggesting a favorable prognosis. Class 2 (“persistent-increase”) started with an initial creatinine value near 1.9 mg/dL, which continued to rise thereafter; this persistently increasing trajectory may indicate progressive deterioration of the patient's overall physical condition, portending a poor prognosis. Class 3 (“transient-increase”) began at approximately 1.7 mg/dL and exhibited a pattern of initial increase followed by a subsequent decrease; this pattern suggests transient physiological insult with subsequent recovery, corresponding to an intermediate-to-favorable prognosis.

The goodness-of-fit statistics for the LCTM are presented in **Table 1**. All models demonstrated high classification accuracy, with entropy values exceeding 0.7. While both AIC and BIC values decreased continuously from the 1-class to the 7-class model, the rate of decline showed a distinct inflection point at the 3-class model. After comprehensive evaluation, the 3-class model was selected as the final model due to its optimal balance of statistical fit and clinical interpretability.

**Table 1:** Fit statistics for different numbers of trajectory groups

Number of classes	Log Likelihood	npm	BIC	AIC	Entropy	Relative entropy	Class 1	Class 2	Class 3	Class 4	Class 5	Class 6	Class 7
1	−4819.19	7	9698.2	9652.38	1	1	100						
2	−315.4544	12	733.458	654.909	581.898	0.837	15.2381	84.7619					
3	212.5419	17	−279.805	−391.084	559.133	0.901	85.67541	9.173955	5.150632				
4	807.6225	22	−1427.238	−1571.245	1470.259	0.794	71.02041	20.97182	2.701652	5.306122			
5	1033.791	27	−1836.845	−2013.582	2237.51	0.73	35.86006	40.71914	16.1516	2.662779	4.606414		
6	1154.722	32	−2035.979	−2245.444	2469.153	0.732	14.55782	34.63557	30.30126	13.60544	2.60447	4.295432	
7	1248.518	37	−2180.842	−2423.036	2641.727	0.736	10.28183	28.41594	30.7483	18.25073	5.597668	2.487852	4.217687

log likelihood: logarithm of the likelihood function; npm: number of free parameters; BIC: Bayesian information criterion; AIC: Akaike information criterion

### Characteristics of creatinine trajectories

**Fig. 2:**
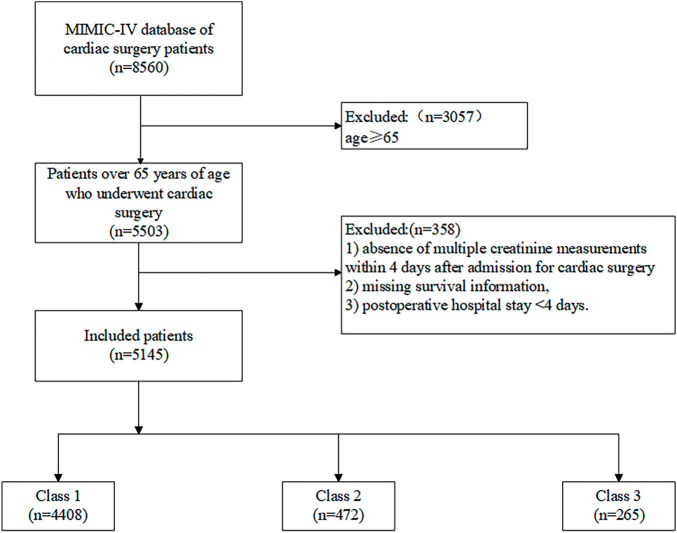
Inclusion and exclusion criteria.

Initially, 8560 patients were screened for eligibility. After applying the inclusion and exclusion criteria, 5145 patients were included in the final analysis. The inclusion and exclusion criteria are shown in **Fig. 2**. All enrolled patients were older than 65 years, with a median age of 75.82. The cohort consisted predominantly of males (3375, 65.6%) and individuals of White ethnicity (3946, 76.7%). Preoperative comorbidities and treatments included myocardial infarction (1546, 30.0%), congestive heart failure (1831, 35.6%), cerebrovascular disease (625, 12.1%), chronic pulmonary disease (1278, 24.8%), and diabetes (1894, 36.8%). During hospitalization, 121 patients (2.4%) received continuous renal replacement therapy, 357 (6.9%) received vasopressin, 93 (1.8%) received dopamine, and 896 (17.4%) received norepinephrine. Extracorporeal circulation was used in 1498 patients (29.1%). **Table 2** illustrates the fundamental characteristics of the aforementioned 3 classes.

**Table 2:** Descriptive characteristics of overall participants and by creatinine trajectory

Variables	Overall (N = 5145)	Group 1 (N = 4408)	Group 2 (N = 472)	Group 3 (N = 265)	p Value
Gender					0.004
Female	3375 (65.6)	2859 (64.9)	319 (67.6)	197 (74.3)	
Male	1770 (34.4)	1549 (35.1)	153 (32.4)	68 (25.7)	
Age (years)	75.82 (6.94)	75.54 (6.87)	77.96 (7.24)	76.55 (6.98)	<0.001
Race					0.017
Other	1199 (23.3)	997 (22.6)	129 (27.3)	73 (27.5)	
White	3946 (76.7)	3411 (77.4)	343 (72.7)	192 (72.5)	
Weight	80.10 [69.00, 92.00]	80.00 [69.00, 91.00]	81.70 [69.90, 95.00]	83.70 [70.80, 98.00]	0.01
Height	170.00 [163.00, 178.00]	170.00 [163.00, 178.00]	170.00 [163.00, 178.00]	170.00 [163.00, 178.00]	0.54
Heart rate	80.00 [73.00, 86.00]	80.00 [73.00, 85.00]	80.00 [74.00, 88.00]	80.00 [73.00, 87.00]	0.005
SBP	112.00 [101.00, 124.00]	112.00 [101.00, 124.00]	109.00 [98.75, 124.00]	110.00 [99.00, 124.00]	0.032
DBP	56.00 [50.00, 64.00]	57.00 [50.00, 64.00]	52.00 [46.00, 60.00]	53.00 [47.00, 60.00]	<0.001
Respiratory rate	15.00 [14.00, 16.00]	15.00 [14.00, 16.00]	16.00 [14.00, 18.00]	16.00 [14.00, 17.00]	<0.001
SpO_2_	100.00 [99.00, 100.00]	100.00 [99.00, 100.00]	100.00 [98.00, 100.00]	100.00 [98.00, 100.00]	<0.001
INR	1.40 [1.20, 1.50]	1.40 [1.20, 1.50]	1.30 [1.20, 1.50]	1.40 [1.20, 1.60]	0.132
PT	14.90 [13.40, 16.60]	15.00 [13.40, 16.50]	14.70 [13.17, 16.72]	15.40 [13.40, 17.00]	0.112
PTT	33.70 [29.00, 49.80]	33.40 [28.90, 48.60]	36.15 [29.90, 59.42]	35.20 [29.50, 56.00]	<0.001
Chloride	108.00 [104.00, 110.00]	108.00 [104.00, 111.00]	106.00 [101.00, 110.00]	107.00 [102.00, 110.00]	<0.001
Bicarbonate	23.00 [22.00, 25.00]	24.00 [22.00, 25.00]	23.00 [21.00, 26.00]	23.00 [21.00, 25.00]	<0.001
BUN	18.00 [14.00, 24.00]	17.00 [14.00, 22.00]	32.50 [21.00, 45.00]	30.00 [22.00, 41.00]	<0.001
Potassium	4.20 [3.90, 4.50]	4.20 [3.90, 4.50]	4.30 [3.90, 4.70]	4.30 [4.00, 4.70]	<0.001
Creatinine	0.90 [0.70, 1.10]	0.90 [0.70, 1.00]	1.70 [1.20, 2.40]	1.50 [1.10, 2.10]	<0.001
WBC	10.20 [7.50, 13.70]	10.20 [7.50, 13.70]	9.40 [7.20, 13.10]	10.70 [7.80, 13.40]	0.028
MCHC	33.30 [32.30, 34.20]	33.30 [32.40, 34.20]	32.70 [31.90, 33.60]	32.80 [31.70, 33.50]	<0.001
RDW	13.80 [13.10, 14.80]	13.70 [13.00, 14.60]	14.70 [13.67, 15.93]	14.40 [13.60, 15.80]	<0.001
RBC	3.17 [2.76, 3.67]	3.19 [2.79, 3.69]	3.06 [2.63, 3.57]	3.04 [2.62, 3.61]	<0.001
Platelet	150.00 [118.00, 195.00]	149.00 [118.00, 194.00]	155.00 [113.75, 203.25]	160.00 [119.00, 207.00]	0.283
MCV	91.00 [88.00, 95.00]	91.00 [88.00, 95.00]	92.00 [88.00, 96.00]	92.00 [88.00, 96.00]	0.014
Hemoglobin	9.60 [8.40, 11.00]	9.70 [8.40, 11.10]	9.20 [8.00, 10.60]	9.00 [7.90, 10.40]	<0.001
Hematocrit	29.00 [25.30, 33.30]	29.10 [25.50, 33.50]	28.35 [24.70, 32.40]	27.80 [24.40, 31.90]	<0.001
Lactate	1.30 [1.00, 1.70]	1.30 [1.00, 1.70]	1.20 [1.00, 1.70]	1.30 [1.00, 1.70]	0.011
pH	7.41 [7.38, 7.44]	7.41 [7.38, 7.44]	7.40 [7.37, 7.44]	7.40 [7.36, 7.43]	<0.001
Oxygenation Index	273.00 [190.00, 367.00]	276.00 [193.00, 369.00]	250.00 [176.75, 355.00]	230.00 [159.00, 336.00]	<0.001
pCO_2_	40.00 [37.00, 44.00]	40.00 [37.00, 44.00]	40.00 [37.00, 44.00]	40.00 [37.00, 44.00]	0.506
Dopamine used					<0.001
No	5052 (98.2)	4354 (98.8)	445 (94.3)	253 (95.5)	
Yes	93 (1.8)	54 (1.2)	27 (5.7)	12 (4.5)	
Vasopressin used					<0.001
No	4788 (93.1)	4170 (94.6)	401 (85.0)	217 (81.9)	
Yes	357 (6.9)	238 (5.4)	71 (15.0)	48 (18.1)	
CRRT					<0.001
No	5024 (97.6)	4389 (99.6)	414 (87.7)	221 (83.4)	
Yes	121 (2.4)	19 (0.4)	58 (12.3)	44 (16.6)	
Delirium					0.003
No	4554 (88.5)	3928 (89.1)	405 (85.8)	221 (83.4)	
Yes	591 (11.5)	480 (10.9)	67 (14.2)	44 (16.6)	
Myocardial infarct					<0.001
No	3599 (70.0)	3164 (71.8)	276 (58.5)	159 (60.0)	
Yes	1546 (30.0)	1244 (28.2)	196 (41.5)	106 (40.0)	
Congestive heart failure					<0.001
No	3314 (64.4)	3008 (68.2)	189 (40.0)	117 (44.2)	
Yes	1831 (35.6)	1400 (31.8)	283 (60.0)	148 (55.8)	
Peripheral vascular disease					<0.001
No	4257 (82.7)	3689 (83.7)	362 (76.7)	206 (77.7)	
Yes	888 (17.3)	719 (16.3)	110 (23.3)	59 (22.3)	
CVD					0.055
No	4520 (87.9)	3892 (88.3)	404 (85.6)	224 (84.5)	
Yes	625 (12.1)	516 (11.7)	68 (14.4)	41 (15.5)	
Dementia					0.308
No	5094 (99.0)	4367 (99.1)	467 (98.9)	260 (98.1)	
Yes	51 (1.0)	41 (0.9)	5 (1.1)	5 (1.9)	
Chronic pulmonary disease					0.044
No	3867 (75.2)	3339 (75.7)	334 (70.8)	194 (73.2)	
Yes	1278 (24.8)	1069 (24.3)	138 (29.2)	71 (26.8)	
Rheumatic disease					0.969
No	4946 (96.1)	4238 (96.1)	454 (96.2)	254 (95.8)	
Yes	199 (3.9)	170 (3.9)	18 (3.8)	11 (4.2)	
Peptic ulcer disease					0.001
No	5105 (99.2)	4382 (99.4)	464 (98.3)	259 (97.7)	
Yes	40 (0.8)	26 (0.6)	8 (1.7)	6 (2.3)	
Mild liver disease					<0.001
No	4979 (96.8)	4295 (97.4)	435 (92.2)	249 (94.0)	
Yes	166 (3.2)	113 (2.6)	37 (7.8)	16 (6.0)	
Diabetes					<0.001
No	3251 (63.2)	2890 (65.6)	224 (47.5)	137 (51.7)	
Yes	1894 (36.8)	1518 (34.4)	248 (52.5)	128 (48.3)	
Diabetes mellitus without complications					0.29
No	3723 (72.4)	3204 (72.7)	327 (69.3)	192 (72.5)	
Yes	1422 (27.6)	1204 (27.3)	145 (30.7)	73 (27.5)	
Diabetes mellitus with complications					<0.001
No	4561 (88.6)	4018 (91.2)	344 (72.9)	199 (75.1)	
Yes	584 (11.4)	390 (8.8)	128 (27.1)	66 (24.9)	
Paraplegia					0.009
No	5073 (98.6)	4354 (98.8)	463 (98.1)	256 (96.6)	
Yes	72 (1.4)	54 (1.2)	9 (1.9)	9 (3.4)	
Renal disease					<0.001
No	3988 (77.5)	3768 (85.5)	134 (28.4)	86 (32.5)	
Yes	1157 (22.5)	640 (14.5)	338 (71.6)	179 (67.5)	
Severe liver disease					0.009
No	5130 (99.7)	4399 (99.8)	469 (99.4)	262 (98.9)	
Yes	15 (0.3)	9 (0.2)	3 (0.6)	3 (1.1)	
Metastatic solid tumor					0.341
No	5123 (99.6)	4391 (99.6)	468 (99.2)	264 (99.6)	
Yes	22 (0.4)	17 (0.4)	4 (0.8)	1 (0.4)	
Extracorporeal circulation auxiliary to open heart surgery					0.11
No	3647 (70.9)	3130 (71.0)	319 (67.6)	198 (74.7)	
Yes	1498 (29.1)	1278 (29.0)	153 (32.4)	67 (25.3)	
Norepinephrine used					<0.001
No	4249 (82.6)	3753 (85.1)	326 (69.1)	170 (64.2)	
Yes	896 (17.4)	655 (14.9)	146 (30.9)	95 (35.8)	

SBP: systolic blood pressure; DBP: diastolic blood pressure; SpO_2_: oxygen saturation; INR: International Normalized Ratio; PT: prothrombin time; PTT: partial thromboplastin time; BUN: blood urea nitrogen; WBC: white blood cell count; MCHC: mean corpuscular hemoglobin concentration; RDW: red cell distribution width; RBC: red blood cell count; MCV: mean corpuscular volume; pH: potential of hydrogen in blood; pCO_2_: partial pressure of carbon dioxide; CRRT: continuous renal replacement therapy; CVD: cerebrovascular disease

### Univariate and multivariate analysis

**Fig. 3:**
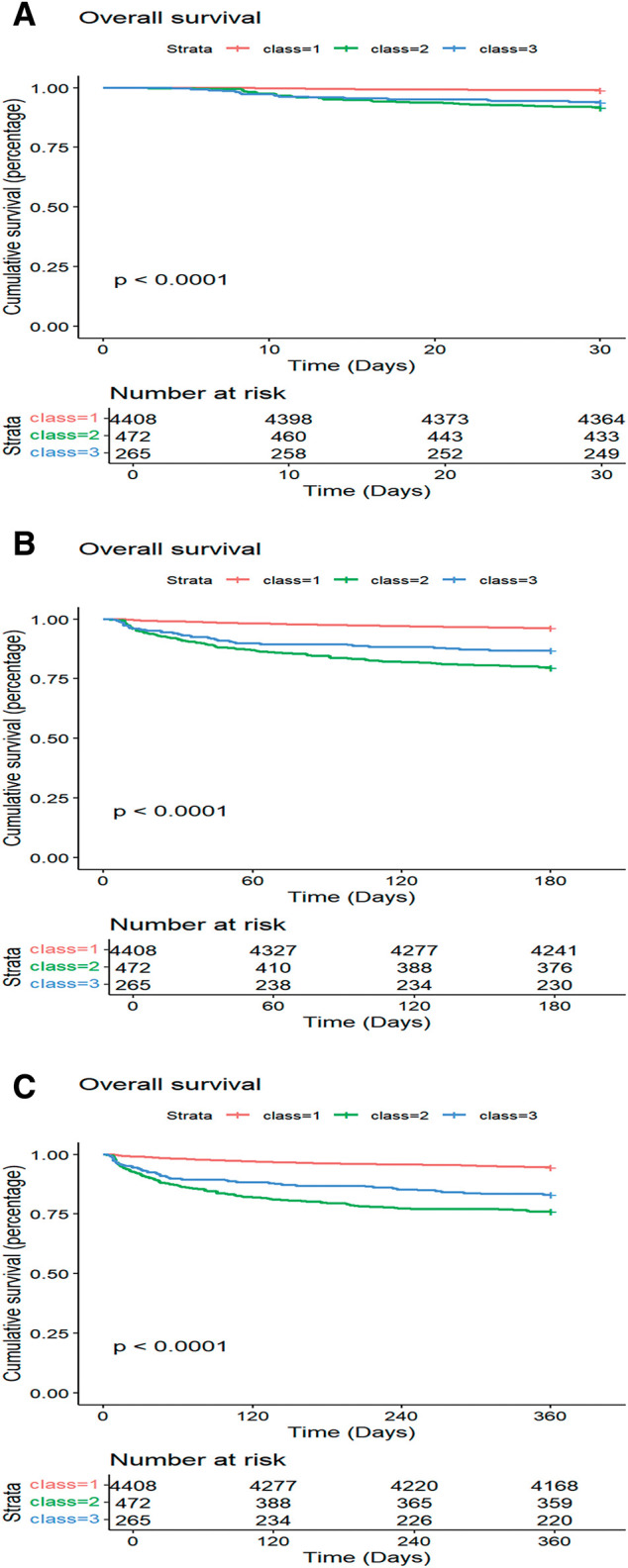
The survival curves were calculated using the Kaplan–Meier method at (**A**) 30 days, (**B**) 180 days, and (**C**) 1 year. Class 1 (“low-stable”) was associated with the lowest mortality, while Class 2 (“persistent-increase”) had the highest mortality

Kaplan–Meier curves comparing survival across the creatinine trajectory classes at 30 days, 180 days, and 1 year are shown in **Fig. 3**. Class 1 demonstrated the lowest 1-year mortality, in contrast to Class 2, which had the highest.

**Table 3** presents the baseline characteristics of 1-year survivors and non-survivors. Based on this univariate analysis, variables significant at p <0.05 were included as covariates in the multivariate Cox proportional hazards model (Model 3).

**Table 3:** Baseline characteristics of the survivors and non-survivors

Variables	Overall	Survival	Non-survivors	p Value
N	5145	4747	398	
Gender				
Female	3375 (65.6)	3157 (66.5)	218 (54.8)	<0.001
Male	1770 (34.4)	1590 (33.5)	180 (45.2)	
Age (years)				
>80	1449 (28.2)	1256 (26.5)	193 (48.5)	<0.001
<=80	3696 (71.8)	3491 (73.5)	205 (51.5)	
Race				
Other	1199 (23.3)	1110 (23.4)	89 (22.4)	0.688
White	3946 (76.7)	3637 (76.6)	309 (77.6)	
Heart rate	80.00 [73.00, 86.00]	80.00 [73.00, 85.00]	81.00 [73.25, 89.00]	<0.001
SBP	113.73 (19.37)	113.62 (19.12)	115.00 (22.16)	0.175
DBP	56.85 (11.07)	57.06 (10.91)	54.33 (12.56)	<0.001
Respiratory rate	15.00 [14.00, 16.00]	15.00 [14.00, 16.00]	16.00 [14.00, 18.00]	<0.001
Hemoglobin	9.60 [8.40, 11.00]	9.60 [8.40, 11.00]	9.30 [8.20, 10.80]	0.007
Creatinine	0.90 [0.70, 1.10]	0.90 [0.70, 1.10]	1.10 [0.90, 1.70]	<0.001
Potassium	4.23 (0.50)	4.22 (0.49)	4.27 (0.56)	0.05
PT	14.90 [13.40, 16.60]	15.00 [13.40, 16.50]	14.80 [13.00, 17.08]	0.01
PTT	33.70 [29.00, 49.80]	33.40 [28.90, 48.40]	41.05 [30.70, 65.33]	<0.001
RBC	3.17 [2.76, 3.67]	3.17 [2.76, 3.67]	3.12 [2.71, 3.67]	0.244
Platelet	150.00 [118.00, 195.00]	149.00 [118.00, 194.00]	163.50 [123.25, 220.75]	<0.001
WBC	10.20 [7.50, 13.70]	10.20 [7.60, 13.70]	9.50 [6.70, 12.80]	<0.001
Lactate	1.30 [1.00, 1.70]	1.30 [1.00, 1.70]	1.30 [1.00, 1.70]	0.022
pH	7.41 (0.05)	7.41 (0.05)	7.40 (0.07)	0.078
pCO_2_	40.61 (6.07)	40.51 (5.75)	41.78 (8.97)	<0.001
CHF				
No	3314 (64.4)	3191 (67.2)	123 (30.9)	<0.001
Yes	1831 (35.6)	1556 (32.8)	275 (69.1)	
Myocardial infarct				
No	3599 (70.0)	3362 (70.8)	237 (59.5)	<0.001
Yes	1546 (30.0)	1385 (29.2)	161 (40.5)	
CVD				
No	4520 (87.9)	4198 (88.4)	322 (80.9)	<0.001
Yes	625 (12.1)	549 (11.6)	76 (19.1)	
Diabetes				
No	3251 (63.2)	3025 (63.7)	226 (56.8)	0.007
Yes	1894 (36.8)	1722 (36.3)	172 (43.2)	

SBP: systolic blood pressure; DBP: diastolic blood pressure; PT: prothrombin time; PTT: partial thromboplastin time; RBC: red blood cell count; WBC: white blood cell count; pH: potential of hydrogen in blood; pCO_2_: partial pressure of carbon dioxide; CHF: congestive heart failure; CVD: cerebrovascular disease

**Fig. 4:**
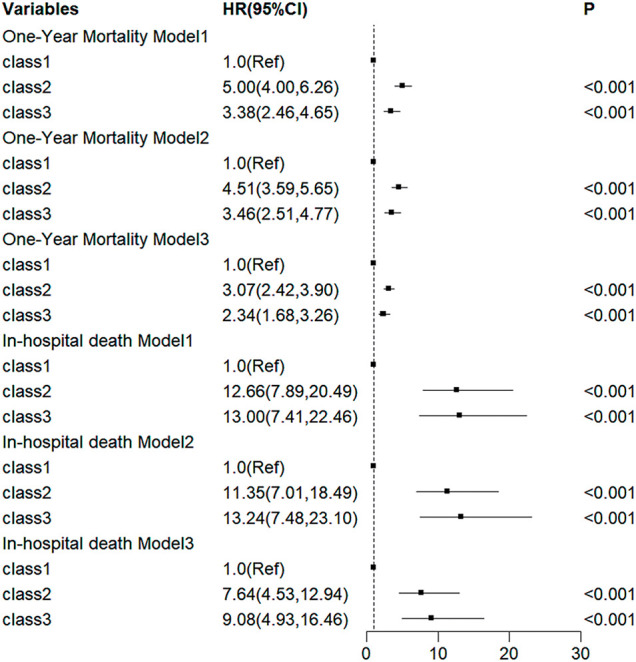
Multivariate Cox proportional hazard analysis was performed using logistic regression analysis to identify risk factors for in-hospital death and 1-year mortality in patients.

The association between creatinine trajectories and patient mortality (in-hospital and 1 year) based on 3 multivariable Cox regression models is shown in **Fig. 4**. Model 1 examined the unadjusted effect of the creatinine trajectory. Model 2 was adjusted for demographic factors (age, sex, race). Model 3 was further adjusted for clinical covariates, including heart rate, respiratory rate, diastolic blood pressure, hemoglobin, prothrombin time, partial thromboplastin time, platelet count, white blood cell count, lactate, partial pressure of carbon dioxide, congestive heart failure, myocardial infarction, cerebrovascular disease, and diabetes. The low-stable class exhibited the lowest mortality risk. Using Class 1 as the reference, Class 2 was associated with the highest hazard risk in the initial model. This association remained consistent even after comprehensive adjustment for potential confounders in Models 2 and 3.

### Subgroup analyses

**Fig. 5:**
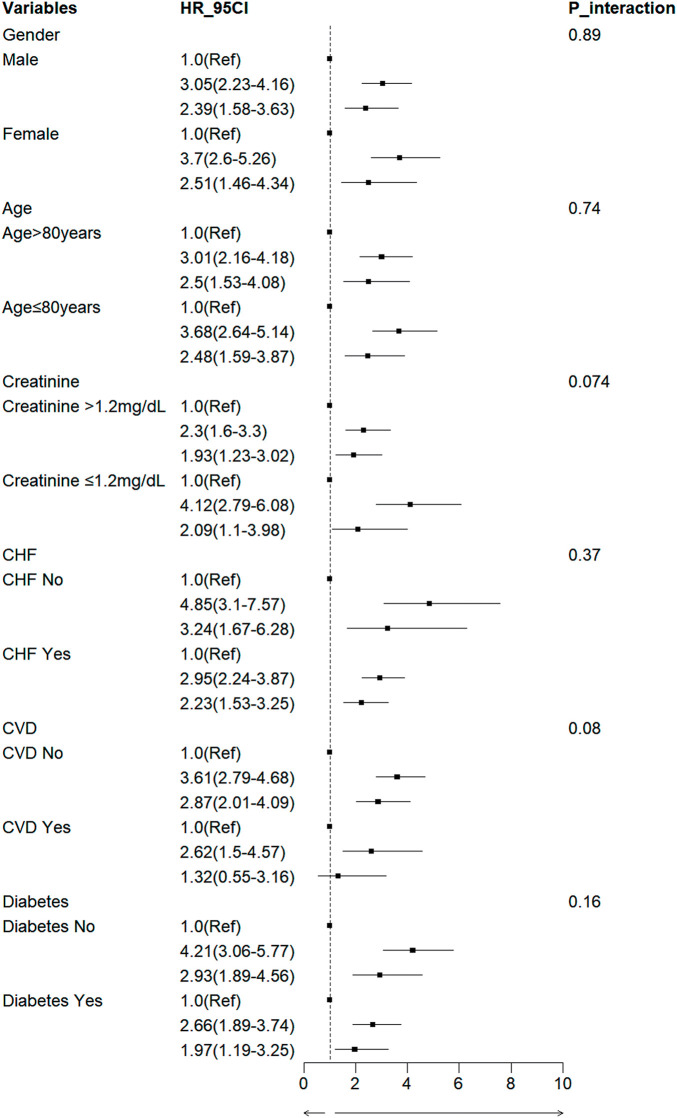
Subgroup analysis using forest plots was performed to explore the relationship between 1-year mortality and creatinine trajectories. CHF: congestive heart failure; CVD: cerebrovascular disease

Subgroup analyses were stratified based on demographic factors (age, sex) and variables identified as significant in the univariate analysis (congestive heart failure, cerebrovascular disease, and diabetes). The forest plot shown in **Fig. 5** illustrates the consistent association across all predefined subgroups; Trajectory 2 was consistently associated with the highest 1-year mortality risk, while Trajectory 1 was associated with the most favorable outcomes. This association remained stable regardless of age class or the presence of specific comorbidities.

## Discussion

This study describes a method for identifying subphenotypes among cardiac surgery patients based on their creatinine trajectories. Through trajectory modeling, we identified and validated 3 distinct subphenotypes that exhibited significant differences in demographic and physiological characteristics, as well as considerable differences in 1-year mortality. The identification of these subphenotypes could facilitate more personalized clinical management strategies.^16)^ This study represents the novel application of trajectory modeling to creatinine dynamics in an elderly cardiac surgery cohort. Our trajectory-based approach offers incremental value by unmasking phenotypic heterogeneity that is not discernible through conventional single-timepoint measurements, thereby enabling early risk stratification and potentially informing more personalized management strategies.

Trajectory-based analysis offers distinct clinical advantages over conventional single-timepoint creatinine assessment. First, while single-timepoint creatinine values merely reflect a patient's physiological status at a specific moment, postoperative creatinine trajectories capture the dynamic evolution of patient conditions over time, enabling the identification of distinct patient subgroups with varying recovery patterns. Notably, the dynamic evolution of creatinine trajectories provides incremental prognostic information beyond static creatinine values: the trajectory pattern (persistent increase versus initial increase followed by subsequent decrease) predicts patient outcomes more robustly than peak creatinine levels alone. For example, patients in Class 2 and Class 3 exhibited comparable baseline creatinine levels, yet demonstrated divergent prognoses—a distinction attributable solely to their subsequent dynamic trajectories rather than to differences in peak values. Second, trajectory analysis reveals actionable time windows for early intervention: creatinine trends within the first 1–4 postoperative days can signal adverse prognosis, providing clinicians with evidence for early risk stratification and timely therapeutic adjustment.

Furthermore, postoperative creatinine trajectories may serve as a surrogate marker of overall disease severity. Patients with persistently elevated postoperative creatinine often exhibit more complicated clinical courses, with underlying pathophysiological factors contributing to poor outcomes. Creatinine trajectories may therefore reflect the cumulative burden of systemic disease processes. Consequently, the prognostic value of creatinine trajectories may partially derive from their capacity to mirror global illness severity.

Subtle fluctuations in serum creatinine levels after surgery can help distinguish between patients with low and high prognostic risks. One study revealed that a decrease in serum creatinine of ≤10% within 6 h after cardiac surgery was associated with a significantly reduced risk of AKI, whereas an increase of ≥10% indicated a substantially elevated risk.^17,18)^ Furthermore, another study demonstrated that an early postoperative rise in serum creatinine was linked to AKI following cardiac surgery, with the area under the receiver-operating characteristic curve being 0.78, indicating good predictive capability.^7)^

Critical illness factors such as fluid overload can contribute to AKI by increasing tubular pressure and reducing glomerular perfusion. In the context of systemic hypoperfusion, AKI may also result from low cardiac output and generalized hypoperfusion due to factors including bleeding, hypovolemia, acute heart failure, or cardiac arrest.^12,19)^ Intraoperative renal perfusion is susceptible to alterations from multiple factors, often resulting in injury to the tubules in the corticomedullary junction and medulla.^20)^ Renal injury can be triggered by ischemia–reperfusion injury, endogenous and exogenous toxins, metabolic disturbances, neurohormonal activation, inflammation, oxidative stress, and hemodynamic instability.^21)^ Elevated serum creatinine and blood urea nitrogen levels may also impair the metabolism of glucose, lipids, proteins, and amino acids, disrupting cellular energy utilization and metabolic homeostasis.^22)^ An increase in creatinine can adversely affect fluid balance, electrolyte stability, drug metabolism, inflammatory responses, and the clearance of metabolic byproducts, thereby influencing renal function and ultimately postoperative survival.^23)^

Hyperglycemia is associated with increased mortality, morbidity, and risk of AKI in patients undergoing cardiac surgery. Maintaining moderate blood glucose levels below 150 mg/dL without resorting to intensive insulin intervention may offer a safer and more effective approach for this patient population.^12,24)^ Advanced age (over 80 years) is an independent predictor of 30-day mortality after aortic valve replacement, with age exerting a significant impact on long-term survival.^25)^ Inotropic and vasopressor agents (e.g., norepinephrine, vasopressin, dopamine) are used to improve renal perfusion and blood pressure during periods of low cardiac output and hypotension. Although vasopressin initially showed promise in treating vasoplegic shock, supporting evidence remains limited^26,27)^; the Acute Dialysis Quality Initiative consensus recommends norepinephrine as the first-line option.^28,29)^ One study also revealed significant sex-based differences in creatinine levels. The proportion of males was higher in the high creatinine class, suggesting a possible influence of sex hormones—testosterone may promote elevated creatinine, whereas estradiol may reduce serum creatinine levels.^30)^

The prognostic role of single-time-point creatinine measurements in the cardiac surgery setting is well documented,^31–34)^ Our study represents a methodological shift by employing LCTM to characterize temporal creatinine dynamics and identify distinct subphenotypes. The subgroup analysis demonstrated the model’s broad applicability, indicating that creatinine trajectory-based stratification can enhance standard clinical evaluations and guideline-directed care by providing dynamic prognostic information.

This study has several limitations. First, the MIMIC-IV database derives from a single US medical center, where perioperative management strategies (e.g., fluid administration, hemodynamic monitoring) and thresholds for renal replacement therapy may differ from those in other regions. Additionally, the exact calendar years could not be extracted due to random temporal shifts, limiting the generalizability of our findings and precluding time-based analyses. Second, the retrospective design inherently cannot fully exclude confounding bias and unmeasured factors (e.g., intraoperative management details), and exclusion criteria may have introduced selection bias; thus, our results primarily demonstrate associations rather than causal relationships. Furthermore, postoperative creatinine is influenced by multiple factors, including hemodilution, fluid balance, and illness severity. However, due to the lack of daily fluid balance, urine output, and dynamic Sequential Organ Failure Assessment scores, we were unable to comprehensively assess true renal function changes or fully evaluate the impact of multi-organ dysfunction; residual confounding may therefore persist. Future multicenter prospective studies incorporating standardized perioperative variables and causal inference methods are warranted to validate these findings.

## Conclusions

This study identified 3 robust and clinically distinct subgroups based on postoperative creatinine trajectories in elderly cardiac surgery patients. A clear gradient in all-cause mortality risk—both in-hospital and long-term—was observed across these trajectories. Patients in the “persistent-increase” class, starting near 1.9 mg/dL, were associated with the highest risk, whereas those in the “low-stable” class, maintaining a level around 0.9 mg/dL, had the most favorable prognosis. This dynamic risk stratification, overlooked in previous studies relying solely on baseline creatinine, demonstrates that creatinine trajectories can identify unique patient subphenotypes and hold significant potential for facilitating personalized clinical management strategies.

## Declarations

### Data availability

The datasets generated and/or analysed during the current study are available in the Medical Information Mart for Intensive Care (MIMIC)-IV repository, [https://mimic.mit.edu/docs/iv/].
